# Supplementary material for: Quantitative Analysis of Hair Luster in a Novel Ultraviolet-Irradiated Mouse Model
Source: Int J Mol Sci. 2024 Feb 4;25(3):1885. doi: 10.3390/ijms25031885 (PMC10855743; doi:10.3390/ijms25031885)
Supplement: Supplementary file 1 [file ijms-25-01885-s001.zip › ijms-2806603-supplementary.pdf]

International Journal of *Molecular Sciences*

MDPI

*Article*

**Supplementary Figures**

# Quantitative Analysis of Hair Luster in a Novel Ultraviolet-Irradiated Mouse Model

**Kyung Bae Chung [1,2,†], Young In Lee [1,2,†], Yoo Jin Kim [3], Hyeon Ah Do [3], Jangmi Suk [3], Inhee Jung [3], Do-Young Kim [1,*] and Ju Hee Lee [1,2,*]**

[1] Department of Dermatology and Cutaneous Biology Research Institute, Yonsei University College of Medicine, Seoul 03722, Republic of Korea; chungkyungbae@yuhs.ac (K.B.C.); ylee1124@yuhs.ac (Y.I.L.)
[2] Scar Laser and Plastic Surgery Center, Yonsei Cancer Hospital, Seoul, Republic of Korea
[3] Global Medical Research Center Co., Ltd., Seoul, Republic of Korea; kyj@gmrc.co.kr (Y.J.K.); dha0201@gmrc.co.kr (H.A.D.); rose@gmrc.co.kr (J.S.); ihjung@gmrc.co.kr (I.J.)
* Correspondence: dykim@yuhs.ac (D.-Y.K.); juhee@yuhs.ac (J.H.L.); Tel.: +82-2-2228-2080 (D.-Y.K. & J.H.L.)
† These authors contributed equally to this work.

**Citation:** Chung, K.B.; Lee, Y.I.; Kim, Y.J.; Do, H.A.; Suk, J.; Jung, I.; Kim, D.-Y.; Lee, J.H. Quantitative Analysis of Hair Luster in a Novel Ultraviolet-Irradiated Mouse Model. *Int. J. Mol. Sci.* **2024**, *25*, 1885. https://doi.org/10.3390/ijms25031885

Academic Editor: Terrence Piva

Received: 19 December 2023
Revised: 30 January 2024
Accepted: 2 February 2024
Published: 4 February 2024

**Copyright:** © 2024 by the authors. Submitted for possible open access publication under the terms and conditions of the Creative Commons Attribution (CC BY) license (https://creativecommons.org/licenses/by/4.0/).

## 1. List of Supplementary Information

### *1.1. Supplementary Figure*

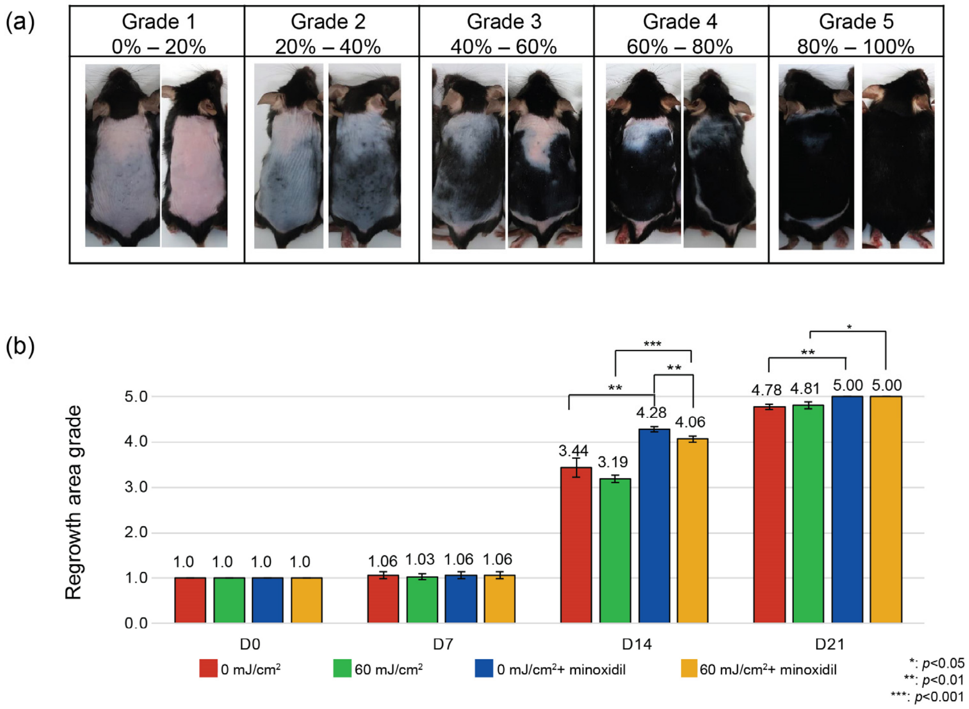


**Figure S1.** Evaluation of growth rate differences induced by UV irradiation (a) Grading scale of growth rate (b) Regrowth area grade evaluation between groups.
